# Urologic, lymphedema, pelvic pain and gastrointestinal symptoms increase after radiotherapy in patients with primary uterine tumors: a prospective longitudinal Swedish cohort study

**DOI:** 10.1007/s12094-021-02576-y

**Published:** 2021-03-08

**Authors:** A. Holmqvist, A. Axelsson, R. Mikivier, K. Redelius, U. Odelram Wiréen, S. Uppugunduri

**Affiliations:** 1 Department of Clinical and Experimental Medicine, Linköping University, Linköping, Sweden; 2 Department of Oncology, Linköping University, S-58185 Linköping, Sweden; 3 Regional Cancer Centre Southeast, Southeast, Sweden

**Keywords:** Uterine tumors, Radiotherapy, Urologic symptoms, Lymphedema symptoms, Pelvic pain, Gastrointestinal symptoms

## Abstract

**Purpose:**

Radiotherapy (RT) causes an inflammatory reaction of the tissue which leads to fibrosis and reduced functioning of the pelvic organs. Few studies have shown significant relationships between side effects and RT in uterine tumors. Here, the urological, lymphedema, pelvic pain and gastrointestinal (GI) symptoms were studied before and after RT in patients with primary uterine tumors using the EORTC QLQ-EN24, specifically designed for uterine cancer patients.

**Methods:**

This prospective cohort study comprised patients with primary uterine tumors who received pelvic radiotherapy (RT). A total of 43 patients were included from May 2014 to February 2019. Patients completed the questionnaires for global health status and functioning before the start of RT and at 3 and 12 months after RT.

**Results:**

We found a significant worsening of the urological symptoms 3 months after RT which persisted up to 12 months after RT compared to baseline values prior to start of RT (*p* = 0.007). An exacerbation of the urinary symptoms was seen in patients with vaginal brachytherapy/boost compared to patients with pelvic RT at 12 months after RT (*p* = 0.053). The severity of lymphedema symptoms increased from RT start to 12 months after RT (*p* = 0.019) and the pelvic pain were higher at 3 months after RT compared to before RT (*p* = 0.004). Also, the level of GI symptoms was significantly higher 12 months after RT compared to the RT start (*p* < 0.001).

**Conclusions:**

The urologic, lymphedema, pelvic pain and GI symptoms all increase after RT.

**Supplementary Information:**

The online version contains supplementary material available at 10.1007/s12094-021-02576-y.

## Introduction

Endometrial cancer is the second most common gynecological cancer in the world and the most common gynecological malignancy in Sweden. Many of these patients are discovered at an early stage and today most of the patients survive their disease. Around 15% of the patients are diagnosed with high-risk disease with an increased risk of distant recurrence and poor survival [1]. For many years, postoperative pelvic external-beam radiotherapy (EBRT) has been the golden standard of treatment for patients with high-risk disease with a significant reduction of loco-regional relapse [2].

External-beam radiotherapy to the pelvis often gives rise to long-term adverse effects that have a significant impact on the patient’s quality of life [3–5]. Radiotherapy (RT) causes an inflammatory reaction of the tissue which leads to fibrosis and reduced function of the pelvic organs. Few prospective studies have found a significant relationship between side effects and pelvic RT in primary uterine tumors.

Previous prospective randomized trials have studied the relationship between urologic symptoms and RT, but found no significant relationships [2, 6–8]. Retrospective studies have shown correlations between lymphedema symptoms and RT [9–12] and pelvic pain and RT [13–15], but no previous prospective study have found significant associations. Gastrointestinal (GI) side effects due to RT are carefully described prospectively [2–6, 8].

Different types of assessment tools have been used to measure symptoms. One study used the Franco-Italian glossary [2] others used the cervical [7, 16, 17], prostate and ovarian cancer modules [6, 8, 18]. As far as we know, no previous study has assessed the symptoms from patients with primary uterine tumors using the EORTC QLQ-EN24 module specifically adapted for uterine cancer patients.

Effective cancer therapies have improved the patient’s survival but have also resulted in a larger number of long-term survivors requiring rehabilitation. Therefore, there is a growing need to identify the type of side effects and the time point at which these side effects may appear in relation to RT.

The aim of this prospective study was to evaluate the type and levels of side effects such as urologic, lymphedema, pelvic pain and GI symptoms in patients with primary uterine tumors using the EORTC QLQ-EN24 form. A secondary aim was to get a better understanding of the incidence and time course of these RT-related side effects.

## Methods

### Patients

The study protocol was approved by the regional ethical committee in Linköping, Sweden (Reference Number: 2018/363–31, 2019/013–33), and was in accordance with the Declaration of Helsinki. All patients had signed a written informed consent form.

This prospective cohort study comprised patients with primary uterine tumors stage I–IV who received pelvic RT. A total of 43 patients were included from May 2014 to February 2019. The patients were treated at the University Hospital of Linköping at the department of gynecological oncology with a catchment-area of ~ 1.5 million people. All patients with primary uterine tumors who received curative doses of pelvic RT (~ 10 patients/year) were asked to participate in the study. Thirty-nine of the 43 patients were in stage 2–3C2 at diagnosis and received postoperative adjuvant RT. Four patients received pelvic RT due to early local recurrence. Three patients were in stage 1A–B and one patient in stage 4B. All patients received treatment according to the national guidelines. The mean age of the patients was 62 years (range 39–76). Patients with dementia, unable to read and speak Swedish, and not capable of receiving the standard adjuvant treatment (co-morbidities and/or poor performance status) were not included in the study.

### Data

Descriptive data such as age, differentiation grade, stage, time of diagnosis, date of surgery, and information about postoperative treatment were obtained from patients’ oncological and surgical records (Table 1).

**Table 1:** Patient characteristics of the 43 primary uterine tumor patients

Variables	*N* (%)
Histopathology	
Endometroid FIGO 1	2 (4.7)
Endometroid FIGO 2	13 (30.2)
Endometriod FIGO 3	13 (30.2)
Serous carcinoma	9 (20.9)
Clear cell carcinoma	1 (2.3)
Mucinous carcinoma	0
Carcinosarcoma	2 (4.7)
Stroma cell carcinoma	2 (4.7)
Leiomyosarcoma	1 (2.3)
Stage	
1A	2 (4.7)
1B	2 (4.7)
2	10 (23.3)
3A	8 (18.6)
3B	7 (16.2)
3C1	5 (11.6)
3C2	8 (18.6)
4A	0
4B	1 (2.3)
Radiotherapy (RT)	
Yes	43 (100.0)
No	0
Type of radiotherapy (RT)	
46 Gy pelvis ± vaginal brachytherapy ± boost primary tumor	33 (76.7)
45 Gy Pelvis/paraaortal ± vaginal brachytherapy ± boost primary tumor	7 (16.2)
50 Gy Pelvis ± boost primary tumor	3 (6.9)
Chemotherapy (CT)	
Yes	35 (81.4)
No	8 (18.6)
Surgery	
^a^High-risk surgery	14 (32.6)
^b^Low-risk surgery	29 (67.4)
Resection margin	
R1	10 (23.3)
R0	33 (76.7)

^a^Total abdominal hysterectomy (TAH), bilateral salpingo-oophorectomy (SOEB), omental resection and pelvic/paraaortic lymphadenectomy

^b^Total abdominal hysterectomy (TAH), bilateral salpingo-oophorectomy (SOEB)

### Quality of life assessment

Two questionnaires the EORTC QLQ-C30 and EORTC QLQ-EN24 were handed out to the patients at baseline (at the first consultation with a radiation oncologist 3–4 weeks before RT start) and then sent home to the patients address at between 3 and 12 months after completion of RT. The EORTC QLQ-C30 is a multidimensional quality of life questionnaire used in clinical trials for cancer patients [19]. The EORTC QLQ-EN24 contains more specific questions concerning the side effects observed in endometrial cancer patients [20]. A response scale from 1 to 4 was used for each item. All subscales responses were converted to 0–100 scales. Higher scores on the symptom scale indicate a higher level of symptoms (i.e. a worse state of the patient), whereas a higher score for the functioning scales/global quality of life assessment represents a better level of functioning (i.e. a better state of the patient). Interpretations of clinically relevant changes were done as described by Cooks et al. (2011) [21].

### Statistics

The non-parametric Wilcoxon signed rank test was used to analyze the scores of symptoms between paired samples before the RT start, at 3 months and 12 months after RT. The Chi-square method and Fischer’s exact test were used to study the differences in frequency of side effects in relation to type of RT treatment. All comparisons were performed using matched cases. Statistical analyses were performed using the software program SPSS version 25 software (IBM, Armonk, NY, USA) and R version 3.5.1. (R Core Team, Vienna, Austria). The tests were two-sided and *p* value of *p* < 0.05 was considered statistically significant.

## Results

### Study population

Fifty patients were invited to complete the EORTC QLQ-C30 and the EORTC QLQ-EN24 questionnaires at RT start. Forty-three (86.0%) of the patients answered the questionnaire at least once. Seven patients (14.0%) did not answer any questionnaires at all. Three of these patients died during enrollment, 1 had a distant recurrence and 3 patients declined to participate. Of the 43 patients, 40 (93.0%) answered the questionnaires before RT, 37 (86.0%) at 3 months and 32 (69.8%) 12 months after RT.

### Treatment

The EBRT was given with 46–50 Gy in 23–25 fractions to the pelvis or 45 Gy in 25 fractions given to the pelvis and para-aortic lymph nodes. Eighteen patients (41.9%) received additional vaginal brachytherapy (2–4 Gy × 4) and 11 (25.6%) patients received a boost to the vagina/parametrium (2 Gy × 6–7). All the treatment was delivered with intensity-modulated radiotherapy (IMRT) (Table 1). The postoperative CT consisted of paclitaxel 175 mg/m^2^ and carboplatin according to the area under the curve (AUC) = 5, given every 3rd week. Thirty-five of 43 patients received adjuvant CT before RT (Table 1).

### Symptom score

**Fig. 1:**
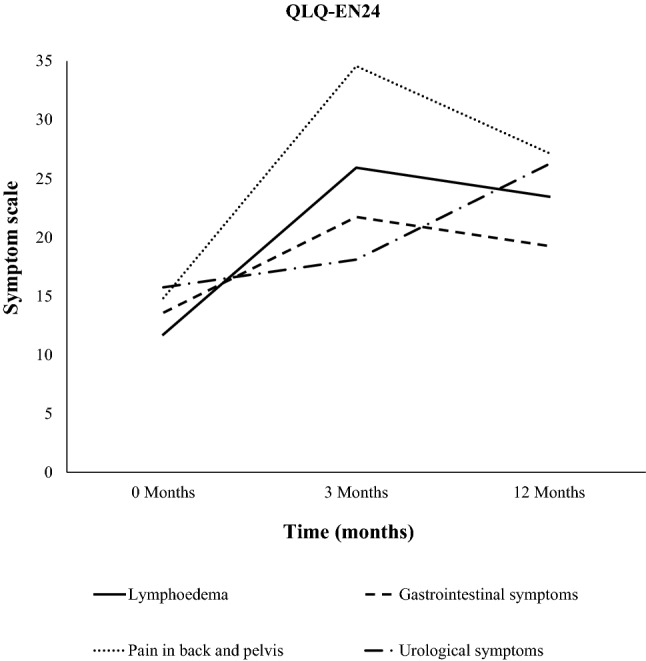
Symptom scoring for urologic, lymphedema, pelvic pain and gastrointestinal (GI) symptoms before RT, 3 months after RT and 12 months after RT in patients with primary uterine tumors

The symptom scores were evaluated using the EORTC QLQ-EN24 in patients with primary uterine tumors with RT. Here, we showed a significant worsening of the urological symptoms 12 months after RT compared to values at RT start (*p* = 0.007, Fig. 1, Table 2). Further, the symptoms of lymphedema increased significantly in severity 12 months after RT compared to before RT (*p* = 0.019, Fig. 1, Table 2). Patients reported increased pain from the pelvic tract 3 months after RT compared to values at RT start (*p* = 0.004, Fig. 1, Table 2). The GI symptoms worsened significantly 12 months after RT compared to the values before RT (*p* = 0.018, Fig. 1, Table 2).

**Table 2:** Data of the global health status, functioning and symptom score from the EORTC QLQ-C30 and QLQ-EN24 questionnaires before RT, 3 and 12 months after RT in patients with primary uterine tumors

Questionnaire, functioning and symptoms	Before RT vs. 3 months after RT	*N*^a^	*p* value	3 months after RT vs. 12 months after RT	*N*^a^	*p* value	Before RT vs. 12 months after RT	*N*^a^	*p-*value
EORTC QLQ-C30 functioning scale									
Global health status	63.7–65.0	34	0.579	68.2–73.1	27	0.232	62.2–71.2	26	0.045
Physical function	79.4–76.5	35	0.137	78.6–83.1	28	0.126	81.2–84.3	28	0.347
Role function	65.7–63.3	35	0.566	68.5–79.2	28	0.044	69.0–78.6	28	0.223
Emotional function	74.8–76.2	34	0.505	79.2–86.0	28	0.013	74.1–85.5	27	0.009
EORTC QLQ-C30 symptom scale									
Diarrhoea	8.8–25.5	34	0.001	23.8–20.2	28	0.366	7.4–22.2	27	0.003
Fatigue	29.7–32.7	35	0.535	32.1–23.6	28	0.535	28.8–22.0	28	0.245
Pain	19.0–24.8	35	0.190	25.0–19.0	28	0.156	20.8–19.0	28	1.000
EORTC QLQ-EN24 symptom scale									
Urological	14.5–16.8	35	0.213	18.1–25.6	28	0.003	15.2–25.3	28	0.007
Lymphedema	12.9–24.3	35	0.007	25.0–22.6	28	0.609	11.3–22.6	28	0.014
Gastrointestinal	13.0–20.8	35	0.001	21.0–18.8	28	0.317	13.3–19.3	28	0.031
Pain in low back/pelvis	14.3–31.4	35	0.006	33.3–26.2	28	0.268	14.3–26.2	28	0.075

^a^All analyses were based on matched cases

### Number of patients with symptoms

Next, we performed a descriptive analysis of symptoms from individual items (Table 3). The proportion of patients with urological symptoms as urinary urgency, frequent visits to the toilet and urinary leakage was at RT start 48.1%, 34.6% and 26.9%, respectively. All of these symptoms increased 12 months after RT to 70.4%, 50.0% and 46.2% (Table 3). The proportion of patients with lymphedema symptoms such as swelling and heaviness at RT start was 18.5% and 23.1% which increased to 51.9% and 50.0% 12 months after RT. The proportion of patients with symptoms of pain in the pelvis increased from 37.0% at RT start to 63.0% 3 months after RT and remained at 55.6% 12 months after RT (Table 3). Finally, the proportion of patients with fecal leakage was 3.7% at RT start and increased up to 22.2% at 12 months after RT. Fecal urgency was present in 63.0% of the patients 12 months after RT (Table 3).

**Table 3:** The percentage of patients with symptoms as; urologic, lymphedema, gastrointestinal and pain in the pelvis before RT, 3 and 12 months after RT in primary uterine tumors

ENGOT EN-24 questionnaire symptoms	*N*^b^	Before RT %^a^	3 months after RT %^a^	12 months after RT %^a^
Urologic symptoms				
When you felt the urge to pass urine, did you have to hurry to get to the toilet?	27	51.9	63.0	70.4
Have you passed urine frequently?	26	38.5	46.2	53.8
Have you had leaking of urine?	26	26.9	34.6	42.3
Have you had pain or a burning feeling when passing urine?	26	19.2	19.2	19.2
Lymphedema symptoms				
Have you had swelling in one or both legs?	27	22.2	48.1	55.6
Have you had heaviness in one or both legs?	26	26.9	50.0	53.8
Gastrointestinal symptoms				
Have you had any leakage of stools?	27	3.7	22.2	22.2
When you felt that you have to empty the bowel, did you need to hurry to visit the toilet?	27	51.9	66.7	59.3
Have you had cramps in your abdomen?	27	29.6	37.0	33.3
Have you had a bloated feeling in your abdomen?	27	44.4	59.3	51.9
Have you been troubled by passing wind?	27	55.6	77.8	55.6
Pain in low back and pelvis				
Have you had pain in your low back or in your pelvis?	27	40.7	63.0	55.6

^a^The number of patients with any grade of symptoms (mild, moderate and severe) presented in percent (%)

^b^All analyses were based on matched cases

### Number of patients with symptoms in relation to RT treatment

The patients with urologic and GI symptoms were further studied in relation to the type of pelvic RT treatment. We found a statistically significant difference in the urologic symptom “urge to pass urine” when patients with pelvic RT were compared with those receiving vaginal brachytherapy/boost at 3 and 12 months after RT (*p* = 0.045, *p* = 0.053). A further subgroup analysis showed that 83.3% of the patients who received brachytherapy, 73.3% with boost and 36.4% of the patients with pelvic RT had symptoms of urinary urgency up to 12 months after RT (*p* = 0.079). No significant differences were found between the type of pelvic RT and GI symptoms after finishing RT (Table 4). The progression-free survival was significantly reduced in the patients with brachytherapy/boost compared to patients with pelvic RT (HR 0.636; CI 95% 1.145–64.68, *p* = 0.034) and the significance remained after correction for age, stage and differentiation grade (HR 0.750; CI 95% 1.172–196.055, *p* = 0.037). A trend towards significance was found for local recurrence-free survival (*p* = 0.071), but no differences were found for distant recurrence-free survival or overall survival (*p* > 0.05).

**Table 4:** Urologic and gastrointestinal (GI) symptoms in relation to the type of RT treatment at 3 and 12 months after RT using the ENGOT EN-24 form

ENGOT EN-24 questionnaire type of RT treatment symptoms	Time		RT *N* (%)	RT + brachytherapy/boost *N* (%)	*p*-value
Urinary symptoms					
When you felt the urge to pass urine, did you have to hurry to get to the toilet?	3 months	Yes	5 (38.5)	18 (72.0)	0.045
		No	8 (61.5)	7 (28.0)	
	12 months	Yes	4 (36.4)	16 (76.2)	0.053
		No	7 (63.6)	5 (23.8)	
Have you passed urine frequently?	3 months	Yes	6 (46.2)	14 (56.0)	0.564
		No	7 (53.8)	11 (40.0)	
	12 months	Yes	6 (54.5)	10 (50.0)	0.809
		No	5 (45.5)	10 (50.0)	
Have you had leaking of urine?	3 months	Yes	3 (23.1)	10 (41.7)	0.258
		No	10 (76.9)	14 (58.3)	
	12 months	Yes	4 (36.4)	9 (42.9)	0.722
		No	7 (63.6)	12 (57.1)	
Gastrointestinal symptoms					
Have you had any leakage of stools?	3 months	Yes	9 (69.2)	14 (56.0)	0.429
		No	4 (30.8)	11 (44.0)	
	12 months	Yes	5 (45.5)	14 (66.7)	0.246
		No	6 (54.5)	7 (33.3)	
When you felt that you have to empty the bowel, did you need to hurry to visit the toilet?	3 months	Yes	2 (15.4)	5 (20.0)	0.549
		No	11 (84.6)	20 (80.0)	
	12 months	Yes	2 (18.2)	5 (23.8)	0.544
		No	9 (81.8)	16 (76.2)	

^a^RT = pelvic and/or paraaortic RT

^**b**^RT + brachytherapy/boost = pelvic and/or paraaortic RT + vaginal brachytherapy/boost to the vagina and/or parametrium

### Patients functioning

**Fig. 2:**
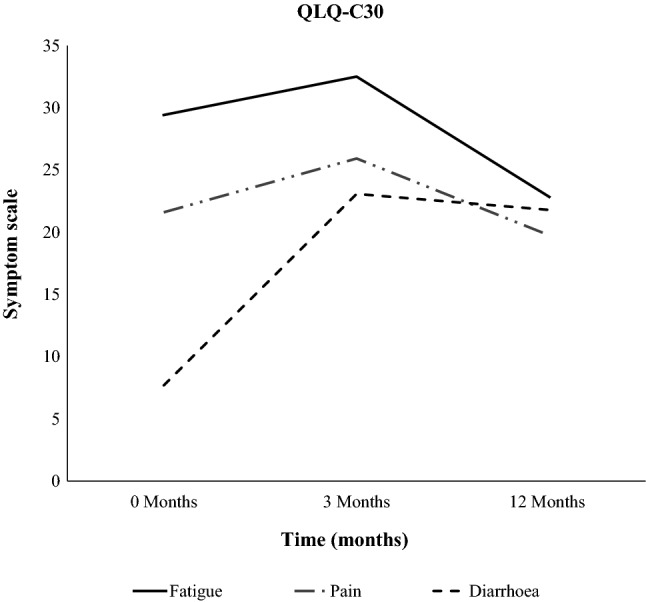
Symptom scoring for fatigue, pain and diarrhea before RT, 3 months after RT and 12 months after RT in patients with primary uterine tumors

**Fig. 3:**
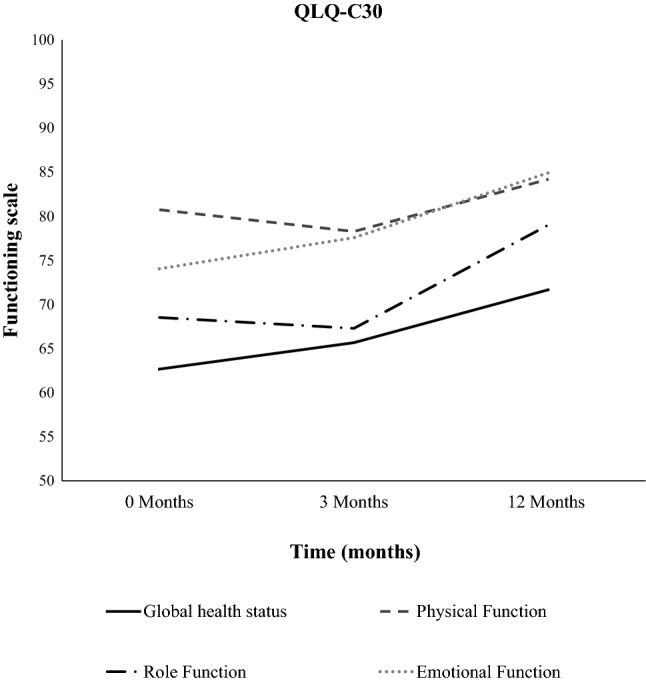
Symptom scoring for general global health status and patients functioning before RT, 3 months after RT and 12 months after RT in patients with primary uterine tumors

We further investigated the global health status and functioning of the patients with primary uterine tumors with RT using the EORTC QLQ-C30. The occurrence of diarrhea was significantly increased 12 months after RT compared to before the start of RT (*p* < 0.001, Fig. 2, Table 2). The scores for role function improved 12 months after RT compared to 3 months after RT (*p* = 0.051, Fig. 3, Table 2). And the scores for emotional function improved 12 months after RT compared to values at RT start (*p* = 0.018, Fig. 3, Table 2).

## Discussion

Radiotherapy causes inflammation and fibrosis of the pelvic tissue which leads to reduced functioning of the pelvic organs that affects the quality of life for many patients. Few prospective studies have shown significant relationships between side effects and pelvic RT in primary uterine tumors.

In this study, we found a significant worsening of the urologic symptoms 12 months after RT compared to values at RT start. We also showed that 70.4% of the patients still had symptoms of urinary urgency, 50.0% had frequent visits to the toilet and 46.2% had urinary leakage 12 months after RT. In previous prospective studies, the frequency of symptoms was lower compared to our study (25.7–32.6%); further, no significant relationship was found between urologic symptoms and RT [6–8]. Urinary symptoms could be caused by damaged nerves and fibrosis initiated by surgery, CT and RT which could lead to an overactive bladder and a reduced ability to preserve urine [22]. The increase in urinary symptoms in our study from RT start to 3 months and then further up to 12 months after RT, might be caused by radiation-induced cystitis but it could also be influenced by the surgery and CT that the patients receive before the start of RT. Interestingly, vaginal boost and brachytherapy induced an exacerbation of urologic symptoms. A majority of the patients receiving vaginal boost or brachytherapy exhibited symptoms of urinary urgency (83.3% and 73.3%, respectively) as compared to 36.4% of the patients receiving pelvic RT only.

Radiation-induced cystitis caused by RT is known to start later, sometimes months to years after RT [23]. Today, symptoms caused by radiation-induced cystitis could be relieved by bladder instillations with chondroitin sulphate or sodium hyaluronate [24, 25]. The urologic symptoms in primary uterine tumors increased significantly with RT and persisted up to 12 months after RT. This is in sharp contrast to all other symptoms studied which subsided with time. A high frequency of urologic symptoms was also found in the patients with vaginal brachytherapy/boost. Also, the risk of recurrence was significantly increased in the brachytherapy/boost group compared to the RT alone group even after adjustment for age, stage and differentiation grade. Our new findings make us suggest that treatment with additional vaginal brachytherapy/boost should be initiated with caution due to the high risk of urologic side effects. We also conclude that the urologic symptoms are underestimated in clinical practice and, therefore, call upon clinicians to be more alert to these symptoms and recommend a referral to a urologist for appropriate diagnosis and treatment.

Lymphedema develops when the lymph vessels are damaged after surgery and RT. Here, we showed that the lymphedema symptoms increased significantly from RT start to 12 months after RT. The proportion of patients with symptoms of swelling and heaviness 12 months after RT was 51.9% and 50.0%, respectively. Few studies have analyzed the relationship between lymphedema symptoms and RT in primary uterine cancer. In previous retrospective studies, a positive correlation was found [9–12]. As far as we know, this is the first prospective study that has demonstrated a significant correlation between lymphedema symptoms and RT. Our results suggest that the symptoms of heaviness and swelling are common after surgery and that these symptoms increase and persist after RT. There are, however, some limitations regarding the methods used to evaluate lymphedema symptoms in our study. Complementary volume measurement of the legs was not performed. The symptoms that the patient’s reports could be due to weight gain or inactivity. Also, this study was prospective, and no randomization was performed regarding surgery. We would therefore recommend that volume measurement and symptom scoring should be performed routinely, at the time for surgery, before RT and with regular follow-ups after RT for early diagnosis and treatment.

Pelvic pain after RT might be caused by micro-fractures and inflammation of the pelvic region [13]. No previous prospective study has analyzed the relationship between pelvic pain and RT in primary uterine tumors. Here, we found a significant increase in pelvic pain from RT start compared to 3 months after RT. We also showed that pelvic pain was present in 37.0% of the patients at the RT start which increased to 63.0% 3 months after RT and more than half of the patients (55.6%) still had symptoms 12 months after RT. Long-term follow-up of gynecologic cancer showed that the number of patients with symptoms varied from 7.8 to 38% [14, 15]. The prevalence of pelvic pain was more frequent in our study compared to others, which could partly be explained by the short follow-up period in our study. Also, the type of RT given in our study could be a contributing factor. Today some of the patients receive complementary high doses of RT to small areas (boost) towards lymph node metastases located close to the pelvic bone/joints which could give rise to more isolated areas of tissue damage and pain. Here, we showed that the pelvic pain caused by RT increases in severity 3 months after RT and then decreases but remains at high levels 12 months after RT. The pelvic pain could be explained by micro-fractures and inflammation in the muscles and joints due to RT. Therefore, early diagnosis with radiological control, referral to a physiotherapist and treatment with anti-inflammatory drugs might help the patient to reduce their symptoms.

Radiotherapy causes chronic changes in the bowel function that have a detrimental effect on the patient’s quality of life [5–8]. We and others have found a significant worsening of the GI symptoms and an increase in the level of diarrhea from the start of RT to 12 months after RT [2, 6, 18]. Further, we showed that 22.2% of the patients still had symptoms of fecal leakage 12 months after RT. The number of patients with fecal urgency was still present in 63.0% of the patients 12 months after RT. No relationship was found between the type of pelvic RT (vaginal brachytherapy/boost vs. pelvic RT alone) and GI symptoms. The GI symptoms are a common problem for patients with primary uterine tumors after RT. The dominating symptoms are diarrhea, fecal leakage and fecal urgency. A more frequent referral to a gastroenterologist for colonoscopy could assist in earlier diagnosis and better treatments.

The patient’s general health functioning was measured by the EORTC QLQ-C30 questionnaire. Here, we showed an increased ability of role functioning and emotional functioning 12 months after RT compared to values at RT start which is in line with previous studies [6–8, 18].

One of the strengths of our study is that it is prospective and longitudinal. We only investigated patients with primary uterine tumors and excluded other types of gynecological cancers. All patients received similar RT treatments with IMRT technique. However, a small sample size is a limitation. Most of the patients in our study received CT before RT compared to other studies where CT was administered after RT which could affect the scores of symptoms [7]. We found several significant relationships regarding side effects which could partly be explained by the fact that, in contrast to other prospective studies, we have used the diagnosis-specific questionnaire EORTC QLQ-EN 24, specially designed for women with endometrial cancer [6–8, 18].

In conclusion, the urologic symptoms in primary uterine tumors increased significantly with RT and persisted up to 12 months after RT. This result was in contrast to all other symptoms studied which decreased with time. Treatment with additional brachytherapy/boost increased the risk of urologic side effects with no clear effect on the risk for recurrence. Early referral to an urologist for cystoscopy for correct diagnosis is strongly recommended. Lymphedema increased with time after RT. Regular volume measurements and symptom scoring should be a part of the routine healthcare. The symptoms of pelvic pain increased after RT. Radiological controls, physiotherapeutic and anti-inflammatory treatment are recommended. GI problems were also common after RT. Early diagnosis with colonoscopy is needed. We recommend further prospective studies with a larger cohort of patients to clarify these issues.

## Supplementary Information

Below is the link to the electronic supplementary material.

Supplementary file1 (DOCX 22 KB)

Supplementary file2 (DOCX 13 KB)
