# Supplementary material for: Urologic, lymphedema, pelvic pain and gastrointestinal symptoms increase after radiotherapy in patients with primary uterine tumors: a prospective longitudinal Swedish cohort study
Source: Clin Transl Oncol. 2021 Mar 8;23(9):1752–60. doi: 10.1007/s12094-021-02576-y (PMC8310482; doi:10.1007/s12094-021-02576-y)
Supplement: Supplementary file 1 — Supplementary file1 (DOCX 22 KB) [file 12094_2021_2576_MOESM1_ESM.docx]

Table 5. The percentage of patients with moderate/severe symptoms before RT, 3 and 12 months after RT in patients with primary uterine tumors.

| **ENGOT EN-24 Questionaire**  **Symptoms** |  |  |  |  |
| --- | --- | --- | --- | --- |
|  | **N**** | **Before RT start**  **%*** | **3 months after RT**  **%*** | **12 months after RT**  **%*** |
|  |  |  |  |  |
| **Urological symptoms** |  |  |  |  |
| When you felt the urge to pass urine, did you have to hurry to get to the toilet? | 29 | 17.2 | 24.1 | 37.9 |
| Have you passed urine frequently? | 28 | 10.7 | 14.3 | 28.6 |
| Have you had leaking of urine? | 28 | 3.6 | 3.6 | 17.9 |
| Have you had pain or a burning feeling when passing urine? | 28 | 3.6 | 0.0 | 7.1 |
|  |  |  |  |  |
| **Lymphedema symptoms** |  |  |  |  |
| Have you had swelling in one or both legs? | 29 | 6.9 | 17.2 | 13.8 |
| Have you had heaviness in one or both legs? | 28 | 14.3 | 21.4 | 14.3 |
|  |  |  |  |  |
| **Gastrointestinal symptoms** |  |  |  |  |
| Have you had any leakage of stools? | 29 | 0.0 | 0.0 | 3.4 |
| Have you been troubled by passing wind? | 29 | 13.8 | 24.1 | 17.2 |
| When you felt that you have to empty the bowel, did you need to hurry to visit the toilet? | 29 | 6.9 | 27.6 | 31.0 |
| Have you had cramps in your abdomen? | 29 | 0.0 | 3.4 | 10.3 |
| Have you had a bloated feeling in your abdomen? | 29 | 0.0 | 3.4 | 3.4 |
|  |  |  |  |  |
| **Pain in low back and pelvis** |  |  |  |  |
| Have you had pain in your low back or in your pelvis? | 29 | 3.4 | 27.6 | 17.2 |
|  |  |  |  |  |

*The percentage of patients with moderate/severe symptoms.

**All data were based on matched cases
