# Supplementary material for: Urologic, lymphedema, pelvic pain and gastrointestinal symptoms increase after radiotherapy in patients with primary uterine tumors: a prospective longitudinal Swedish cohort study
Source: Clin Transl Oncol. 2021 Mar 8;23(9):1752–60. doi: 10.1007/s12094-021-02576-y (PMC8310482; doi:10.1007/s12094-021-02576-y)
Supplement: Supplementary file 2 — Supplementary file2 (DOCX 13 KB) [file 12094_2021_2576_MOESM2_ESM.docx]

| **Diseases and medications** | **N = 43 (%)** |
| --- | --- |
| **Other diseases** |  |
| Yes | 24 (55.8) |
| No | 19 (44.2) |
| **Type of diseases** |  |
| Hypertonia | 19 (44.1) |
| Hyperlipidemia | 7 (16.3) |
| Diabetes | 6 (14.0) |
| Other cancers | 5 (11.6) |
| Cardiovascular diseases | 5 (11.6) |
| Reumatic disease | 3 (7.0) |
| Tromboembolic disease | 2 (4.7) |
| **Other medications** |  |
| Blood pressure medications | 17 (39.5) |
| Statins | 7 (16.3) |
| Acetylsalicyclic acid | 5 (11.6) |
| Anticoagulants | 5 (11.6) |
| Diuretics | 4 (9.3) |
| Antidiabetic medications | 4 (9.3) |
| Folic acid | 2 (4.7) |
| Steroids | 1 (2.3) |
|  |  |

Table 6. Other diseases and medications at the time for diagnosis of the 43 primary uterine tumor patients
